# Assessing health-related quality of life in urology – a survey of 4500 German urologists

**DOI:** 10.1186/s12894-017-0235-1

**Published:** 2017-06-19

**Authors:** A. Schmick, M. Juergensen, V. Rohde, A. Katalinic, A. Waldmann

**Affiliations:** 1 Institute for Social Medicine and Epidemiology, University Luebeck, Ratzeburger Allee 160 (Hs 50), 23562 Luebeck, Germany; 2 Department of Emergency Medicine, Klinik Hirslanden, Witellikerstrasse 40, 8032 Zurich, Switzerland; 3 Institute for History of Medicine and Science Studies, University Luebeck, Koenigstr. 42, 23552 Luebeck, Germany; 4 Medical Practice of Urology, Auguststr. 4, 23611 Bad Schwartau, Germany; 5 Institute for Cancer Epidemiology, University Luebeck, Ratzeburger Allee 160 (Hs 50), 23562 Luebeck, Germany

**Keywords:** Health-related quality of life, Assessment, Urology, Survey

## Abstract

**Background:**

Urological diseases and their treatment may negatively influence continence, potency, and health-related quality of life (HRQOL). Although current guidelines recommend HRQOL assessment in clinical urology, specific guidance on how to assess HRQOL is frequently absent. We evaluated whether and how urologists assess HRQOL and how they determine its practicality.

**Methods:**

A random sample of 4500 (from 5200 identified German urologists) was drawn and invited to participate in a postal survey (an initial letter followed by one reminder after six weeks). The questionnaire included questions on whether and how HRQOL is assessed, general attitudes towards the concept of HRQOL, and socio-demographics. Due to the exploratory character of the study we produced mainly descriptive statistics. Chi^2^-tests and logistic regression were used for subgroup-analysis.

**Results:**

1557 urologists (85% male, with a mean age of 49 yrs.) participated. Most of them (87%) considered HRQOL assessment as ‘important’ in daily work, while only 7% reported not assessing HRQOL. Patients with prostate carcinoma, incontinence, pain, and benign prostate hyperplasia were the main target groups for HRQOL assessment. The primary aim of HRQOL assessment was to support treatment decisions, monitor patients, and produce a ‘baseline measurement’. Two-thirds of urologists used questionnaires and interviews to evaluate HRQOL and one-quarter assessed HRQOL by asking: ‘How are you?’. The main barriers to HRQOL assessment were anticipated questionnaire costs (77%), extensive questionnaire length (52%), and complex analysis (51%).

**Conclusions:**

The majority of German urologists assess HRQOL as part of their clinical routine. However, knowledge of HRQOL assessment, analysis, and interpretation seems to be limited in this group. Therefore, urologists may benefit from a targeted education program.

**Trial registration:**

The clinical trial was registered with the code VfD_13_003629 at the German Healthcare Research Registry (www.versorgungsforschung-deutschland.de).

## Background

The most widely-accepted definition of health-related-quality-of-life (HRQOL) is that from the World Health Organization (WHO), which organisation defines this as an ‘individual’s perception of their position in life in the context of the culture and value systems in which they live and in relation to their goals, expectations, standards and concerns’. Consequently, physical and mental health, the measure of one’s independence, social relationships and spiritual/religious beliefs influence the broad concept of HRQOL [1].

Over the last few decades, standardized generic and disease-specific instruments for the assessment of HRQOL have been developed and widely used in research – i.e. HRQOL has been commonly used as a clinical endpoint of therapy-comparing studies. Furthermore, HRQOL has recently gained increased relevance in clinical practice as the guidelines of most medical associations consider the enhancement of HRQOL as one of the primary therapeutic endpoints.

To the best of our knowledge, only a few studies have explored the view of clinicians regarding the concept of HRQOL and its potential for the clinical routine. In an earlier survey, 154 oncologists were interviewed about their attitudes toward HRQOL assessment. While 93.5% of participants reported being familiar with HRQOL research, 64% had assessed HRQOL for research purposes. Moreover, while 28% had used standardized questionnaires, 20% preferred self-made instruments. The research group identified the latter as patient-reported-outcome (PRO) scales [2]. Thus, the difference between HRQOL and patient-reported-outcome measures (PROMs) may not have been clear.

Another group surveyed 89 data managers from the EORTC-Trials regarding their attitude towards HRQOL assessment and found that not only financial and time resources, but also insufficient knowledge about the concept were substantial barriers to the integration of HRQOL assessment in clinical routine [3].

A review of older studies suggests that HRQOL is rarely measured in clinical settings due to a lack of financial and time resources, bureaucratic effort, and insufficient methodical knowledge (know-how) of HRQOL assessment [4].

A more recent survey of 309 Italian clinicians reported that 73.5% would like to assess HRQOL and 94.3% would be willing to prescribe expensive drugs to increase HRQOL. Again, the barriers to doing this were cited as insufficient methodical knowledge accompanied by restrained financial and time resources. Nonetheless, the number of clinicians who routinely measured HRQOL remains unclear [5].

Most studies on HRQOL assessments have investigated relatively small populations of oncologists [2–4]. The study participants were often familiar with HRQOL research and so they may not have been representative. Some of them did not differentiate between PROs and HRQOL [2].

The guidelines of the German Society of Urology (DGU) declare HRQOL as the principal therapeutic aim [6]. Nevertheless, many researchers continue to question the importance of HRQOL in clinical urology [7]. To our knowledge, thus far no studies have been conducted to explore physicians’ proficiency in and attitudes towards HRQOL. Furthermore, the methods of HRQOL assessment in clinical urology remain uncertain.

The objectives of this study are to determine the importance of HRQOL in the clinical setting and to survey how useful, comprehensive, and accessible clinicians estimate HRQOL assessment to be. Moreover, we aim to ascertain the patient cohorts where HRQOL is frequently measured and to assess which methods and HRQOL instruments are regularly applied.

## Methods

### Questionnaire development

In numerous successive expert meetings (A.W., V.R, A.K.) we developed the survey and, to test its usability, conducted two subsequent pre-tests, improving the design after the first pre-test (*n* = 16) and, due to satisfactory results, finalizing it following the second pre-test (*n* = 10).

The final survey consisted of three parts:

(1) 15 closed questions about the attitude towards HRQOL (Likert-scales),

(2) eight questions concerning the assessment of HRQOL in clinical routine (30 items in total for multiple choice questions and extra space for comments),

(3) demographic data (year of birth, year of specialty certification, working environment) and the last two items from the EORTC-QLQ-C30 to assess the HRQOL of the study participants [8–10] as well as extra space for comments.

### Postal survey

We conducted a cross-sectional, nationwide postal survey of German urologists. The addresses were previously obtained from the register of the Association of the Statutory Health Insurance for Physicians and the German Association of Urologists. Our financial resources were capable of covering a survey of 4500; as such, after the revision of the database, a random sample of 4500 out of 5200 urologists was drawn.

The questionnaire was sent out with a post-paid return envelope. We identified non-respondents and sent them a reminder, containing the survey, six weeks after the initial dispatch.

### Statistics

We hypothesized that the HRQOL played an essential role in clinical practice if more than 30% of participants were employing validated questionnaires for recorded HRQOL assessment.

Besides the hypothesis, our study had a mainly exploratory character and we produced primarily descriptive statistics. Additionally, to provide finer distinctiveness, Likert-scale items were added as follows: ‘absolutely disagree’ and ‘slightly disagree’ were condensed into ‘disagree’; consequently, ‘fairly agree’ and ‘absolutely agree’ were subsumed into the category ‘agree’. One question had a different scale so that ‘absolutely not important’ and ‘slightly important’ became ‘not important’, ‘fairly important’, ‘very important’ and ‘important’, while ‘more or less important’ was not included in either category.

Participants were divided into subgroups by gender, age, working environment and status of specialty training to investigate possible group differences. The subgroup analysis was calculated using Chi-square tests and logistic regression. The complete data analysis was accomplished using SPSS 20.0 software.

## Results

### Sample description

We contacted 4500 German urologists. The response rate was 37.9%. Accordingly, there were no statistically significant differences between the socio-demographics of respondents and non-respondents.

The mean age of respondents was 49 years (SD: 9.8) and a significant majority was male (85%). Slightly more than half of them were working in private practices (55.3%) and, consequently, most respondents had completed their specialty training in urology (94.6%; Table 1).

**Table 1:** Description of study participants

	Female *n* = 239	Male *n* = 1318	Total *n* = 1557
Age^a^			
Mean age in years (SD) Range	43.8 (8.1) 31–72	50.4 (9.8) 27–90	49.4 (9.8) 27–90
Consultant			
Consultants (%)	88.3	95.8	94.6
Number of years as Consultant (mean (SD))^b^	11 (8.2)	17 (10.0)	16.2 (10.0)
Working Environment^c^			
Private Practice (%) [eigene Niederlassung]^d^	19.8	32.0	30.1
Group (private) Practice (%)[Gemeinschaftspraxis]^d^	20.3	26.1	25.2
Certified Prostate Centers (%)[zertifiziertes Prostatazentrum]^d^	6.9	8.7	8.4
District hospital (%) [Maximalversorger]^5^	34.9	20.0	22.3
Community hospital (%) [Schwerpunktversorger]^d^	15.1	16.3	16.1
General hospital (%) [Regelversorger]^d^	12.9	12.4	12.5

^a^6 (4 females and 32 males) have not provided their age

^b^118 (34 females und 84 males) have not provided the year of their consultant exam

^c^Percentages based on 1.515 due to missing information

^d^German translation

### Attitude towards HRQOL

#### Relevance of HRQOL assessment in the clinical routine

HRQOL assessment was recognized as an important part of the clinical routine by most urologists (86.5%; Table 2). The perceived importance was reported more frequently with ascending age in respondents (*p* = 0.124, Chi^2^-test). Consequently, consultants venerated HRQOL assessments more than urologists in training (*p* = 0.009, Chi^2^-test). Nevertheless, gender and workplace did not significantly influence the perceived value of HRQOL assessment in the clinical routine.

**Table 2:** Attitudes towards HRQOL assessment

Clinical Importance		Not important		Important		
		Absolutely not important	Slightly important	Fairly important	Very important	More or less important
Is HRQOL assessment important for clinical work?	% (N)	0.3 (5)	2.0 (29)	44.8 (659)	41.7 (614)	11.2 (165) [9.6–12.8]
	%_∑_	2.3 [1.5–3.1]		86.5 [84.7–88.3]		
Perception of HRQOL		Disagree		Agree		
		Absolutely disagree	Slightly disagree	Fairly agree	Absolutely agree	Cannot estimate
To me HRQOL is a vague term.	% (N)	0.6 (9)	5.4 (84)	29.2 (453)	64.0 (992)	0.7 (11) [0.3–1.1]
	%_∑_	6.0 [4.8–7.2]		93.3 [92.1–94.5]		
The difference between HRQOL assessment and symptom rating is not apparent.	% (N)	55.1 (849)	32.4 (499)	8.0 (123)	2.7 (42)	1.9 (29) [1.2–2.6]
	%_∑_	87.4 [85.7–89.1]		10.7 [9.2–12.2]		
I regard HRQOL assessment as not suitable for daily use.	% (N)	15.7 (243)	46.4 (717)	28.1 (435)	5.5 (85)	4.3 (66) [3.3–5.3]
	%_∑_	62.1 [59.7–64.5]		33.6 [31.2–36.0]		
Integrity of HRQOL						
HRQOL assessments are valuable in patient consultations.	% (N)	0.5 (8)	3.3 (51)	23.6 (366)	71.2 (1.103)	1.4 (22) [0.8–2.0]
	%_∑_	3.8 [2.8–4.8]		94.8 [93.7–95.9]		
HRQOL assessments are valuable in therapy follow-ups.	% (N)	0.9 (14)	2.8 (43)	24.2 (375)	71.2 (1.106)	0.9 (14) [0.4–1.4]
	%_∑_	3.7 [2.8–4.6]		95.4 [94.4–96.4]		
Verbal HRQOL assessment is generally sufficient.	% (N)	5.9 (92)	37.7 (585)	36.1 (559)	19.1 (296)	1.2 (18) [0.7–1.7]
	%_∑_	43.6 [41.1–46.1]		55.2 [52.7–57.7]		
Validated HRQOL instruments are useful for HRQOL assessment.	% (N)	2.7 (42)	21.7 (337)	32.9 (511)	39.5 (613)	3.2 (50) [2.3–4.1]
	%_∑_	24.4 [22.3–26.5]		72.4 [70.2–74.6]		
Barriers for HRQOL assessment						
My patients do not accept HRQOL questionnaires.	% (N)	36.0 (558)	43.6 (675)	10.0 (155)	2.7 (41)	7.7 (120) [6.4–9.0]
	%_∑_	79.6 [77.6–81.6]		12.7 [11.0–14.4]		
I prefer not to pay for HRQOL questionnaires.	% (N)	5.0 (77)	5.8 (90)	13.1 (203)	64.1 (989)	12.0 (185) [10.4–13.6]
	%_∑_	10.8 [9.3–12.3]		77.2 [75.1–79.3]		
The effort is too extensive to assess HRQOL in clinical routine.	% (N)	10.4 (161)	37.7 (585)	34.7 (538)	16.2 (251)	1.0 (16) [0.5–1.5]
	%_∑_	48.1 [45.6–50.6]		50.9 [48.4–53.4]		
HRQOL questionnaires are disadvantageous due to their length.	% (N)	4.2 (66)	23.5 (363)	36.8 (569)	15.5 (240)	20.0 (309) [18.0–22.0]
	%_∑_	27.7 [25.5–29.9]		52.3 [49.8–54.8]		
HRQOL questionnaires are disadvantageous due to the complexity of their interpretation.	% (N)	5.2 (81)	25.0 (386)	34.2 (528)	13.9 (214)	21.7 (335) [19.6–23.8]
	%_∑_	30.2 [27.9–32.5]		48.1 [45.6–50.6]		
I am not sufficiently trained to assess HRQOL.	% (N)	39.7 (617)	46.5 (722)	9.6 (149)	1.9 (29)	2.3 (36) [1.6–3.0]
	%_∑_	86.2 [84.5–87.9]		11.5 [9.9–13.1]		
I cannot invoice HRQOL assessment due to a missing number in the medical-fee schedule.	% (N)	6.9 (105)	5.5 (84)	8.0 (122)	40.7 (619)	38.9 (593) [36.5–41.3]
	%_∑_	12.4 [10.7–14.1]		48.7 [46.2–51.2]		

%_∑_ = composite score in percent

[…] = 95% CI

#### Perception of HRQOL

The statement ‘HRQOL is a vague term’ was acknowledged by the majority of doctors (93.3%, Table 2). Moreover, private practice urologists approved the statement more frequently than those from hospitals (OR = 1.77; 95% CI: 1.10–2.83).

Furthermore, the difference between symptom rating and HRQOL assessment was apparent for most of the respondents (87.9%). With increasing age, however, this difference became slightly less definite (OR = 0.97, 95% CI: 0.96–0.99).

Consequently, HRQOL assessments were considered suitable for daily use by more than 62 % (62.1%, Table 2). Notwithstanding, doctors from private practices found HRQOL assessments less suitable for everyday use than those doctors working in hospitals (OR = 1.46; 95% CI: 1.14–1.86).

#### Integrity of HRQOL in clinical routine

Numerous physicians deemed HRQOL assessments as valuable in consultations (94.8%) and therapy follow-ups (95.4%; Table 2). Additionally, verbal HRQOL assessment was considered sufficient by slightly more than half of the physicians (55.2%). Moreover, urologists from private practices (OR = 3.05; 95% CI: 2.40–3.87) preferred verbal HRQOL assessment compared to those from hospitals.

Concurrently, almost three-quarters of physicians approved standardized measures for HRQOL assessment as useful (72.4%, Table 2), whereas urologists occupied in private practices, and those advancing in age, reported the use of validated instruments less frequently (age: OR = 0.98; 95% CI 0.96–0.99 / private practice: OR = 0.37; 95% CI: 0.28–0.49).

Predominantly, urologists stated that their patients would ordinarily accept questionnaires for HRQOL assessment (87.3%). Notwithstanding, patients approved HRQOL surveys in private practices less often (OR = 0.53; 95% CI: 0.37–0.76) than patients in hospitals.

### Barriers to HRQOL assessment

The payment for HRQOL questionnaires was regularly considered inadmissible (77.2%, Table 2). Half of the physicians considered the effort of HRQOL assessment as too voluminous (50.2%). Furthermore, urologists from private practices reported this more frequently (OR = 1.43; 95% CI: 1.14–1.80) than those working in hospitals.

The HRQOL questionnaires were regarded by half of the physicians as disadvantageous due to their length (52.3%) and the complexity of their interpretation (48.1%). Both disadvantages were reported more frequently by physicians in private practice (length: OR = 1.50; 95% CI: 1.14–1.97 / complexity: OR = 1.64; 95% CI: 1.26–2.19) compared with their colleagues from hospitals.

A significant number of urologists regarded themselves to be adequately trained to assess HRQOL (86.2%). Conversely, those from private practices considered themselves less sufficiently trained (OR = 0.62; 95% CI: 0.44–0.88) than those doctors from hospitals.

Most urologists said it was impossible to invoice the HRQOL assessment due to a missing number in the medical-fee schedule (79.7%, Table 2). Nonetheless, this has been our survey’s most frequently unanswered question. Consequently, private practice physicians tended to answer it twice as often (OR = 2.32; 95% CI: 1.60–3.37) when compared with hospital doctors.

### Clinical implementation of HRQOL

The second part of our survey examined the clinical implementation of HRQOL. Almost every urologist assessed HRQOL (93.5%). There were no differences between subgroups.

#### Patient cohorts

**Fig. 1:**
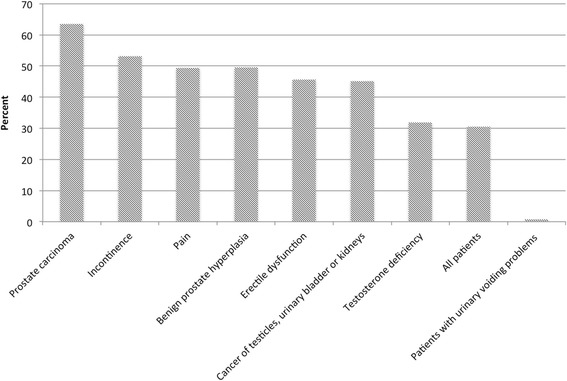
Frequency of HRQOL assessment in different patient groups, according to diagnosis

Urologists most frequently assessed HRQOL in prostate cancer patients (63.5%) followed by those with incontinence (53.2%). Conversely, only a few doctors assessed HRQOL in patients with testosterone deficiency (31.8%; Fig. 1).

#### Aims for HRQOL assessment

The motivation for HRQOL assessment was primarily to support a therapy choice (82.8%), evaluate a follow-up (82.1%) and survey a baseline (75.2%). Urologists rarely assessed HRQOL for research purposes (17.2%).

#### Methods of HRQOL-assessment

Most urologists used combined recorded and verbal HRQOL assessment (61.8%), followed by verbal-only (22.0%) and written-only (10.8%). Consequently, female urologists used the combined approach more regularly (75,9% vs. 64,7%, *p* = 0,003; Chi^2^-Test). Nevertheless, males, private practice urologists, and consultants favored the verbal-only assessment (Table 3).

**Table 3:** Methods of HRQOL assessment in subgroup analysis (Chi^2^-Test and logistic regression analysis)

HRQOL assessment	Recorded and verbal Cells-% (N)	Verbal only Cells-% (N)	Recorded only Cells-% (N)	“How are you?” Cells-% (N)	Verbal Standardized HRQOL Instruments Cells-% (N)	Recorded Non- Standardized Cells-% (N)	Recorded Standardized HRQOL Instruments		
							Cells-% (N)	Multivariate OR^a^ [95% CI]	Multivariate Significance^a^ (Logistic Regression)
Total	61.8 (960)	22.0 (342)	10.8 (143)	53.7 (833)	20.6 (320)	13.5 (209)	65.9 (1023)		
Gender									
Male	64.7^b^ (790)	24.6^b^ (300)	10.7^b^ (131)	52.5^e^ (690)	20.7 (272)	13.0 (171)	65.2 (857)	1	*P* = 0.033
Female	75.9 (170)	18.8 (42)	5.4 (12)	59.8 (143)	20.1 (48)	15.9 (38)	69.5 (166)	0.54 [0.31–0.95]	
Working Environment									
Hospital	63.3^c^ (433)	12.5^c^ (81)	18.1^c^ (117)	48.2^c^ (432)	17.3^c^ (126)	12.8 (89)	77.6^c^ (541)	1	*P* = 0.178
Private practice	69.3 (448)	33.8 (231)	2.9 (20)	5.2 (336)	24.5 (170)	14.2 (104)	55.9 (408)	0.64 [0.29–1.44]	
Age									
25-44y	74.1^c^ (386)	13.4^c^ (70)	12.5^c^ (65)	56.6 (318)	24.4^f^ (137)	12.3 (69)	77.6^c^ (436)	0,96^h^ [0.93–0.98]	*P* < 0.001^h^
45-59y	64.2 (433)	26.9 (181)	8.9 (60)	52.9 (379)	18.7 (134)	13.8 (99)	62.8 (450)		
60-90y	55.0 (120)	38.5 (84)	6.4 (14)	50.0 (121)	16.9 (41)	14.5 (35)	47.5 (115)		
Qualification									
Consultant	66.4 (909)	24.1^d^ (330)	9.4^d^ (129)	53.3 (785)	20.5 (302)	13.5 (199)	65.3^g^ (962)	1	*P* = 0.981
In Training	66.2 (51)	15.6 (12)	18.2 (14)	59.3 (48)	22.2 (18)	12.3 (10)	76.5 (62)	0.99 [0.34–2.88]	

^a^controlled for gender, working environment, age and qualification

^b^ *p* = 0.003 Chi^2^-Test

^c^ *p* < 0.001 Chi^2^-Test

^d^ *p* = 0.019 Chi^2^-Test

^e^ *p* = 0.036 Chi^2^-Test

^f^ *p* = 0.014 Chi^2^-Test

^g^ *p* = 0.038 Chi^2^-Test

^h^linear age model

The oral HRQOL assessment was frequently reduced to the single question: ‘How are you?’ (53.7%), and it was rarely carried out with validated questionnaires in the sense of a structured interview (20.6%). Private practice and female physicians tended to assess HRQOL by asking: ‘How are you?’, more commonly. Conversely, urologists from hospitals and those of a younger age preferred validated questionnaires for verbal HRQOL assessment (Table 3).

More than 65 % of urologists (65.9%) applied validated questionnaires for recorded HRQOL-assessment. Notwithstanding, female urologists tended to use validated questionnaires less often (OR = 0.54; 95% CI: 0.31–0.95). Moreover, with advancing age the probability of validated questionnaire use slightly decreased (OR = 0.96, 95% CI: 0.93–0.98).

Urologists most frequently applied the International Prostate Symptom Score (IPSS) (96.1%) followed by the International Index of Erectile Function (IIEF) (78.7%), the Karnofsky-Index (44.7%), and Aging Male Symptoms (AMS) (36.8%). Conversely, the Eastern Cooperative Oncology Group Score (ECOG) was infrequently used (1.7%). Surprisingly, the EORTC-QLQ-C30, which is recommended by the German Society of Urology for HRQOL assessment in prostate-cancer-patients, was administered rather scarcely (4.5%). Other rarely-used scores included the International Consultation on Incontinence Questionnaire (ICIQ) (4.1%) and the International Continence Society on Quality of Life (ICSQoL) (1.8%). In a bivariate analysis, younger physicians preferred the IPSS (*p* < 0.001, Chi^2^-Test). Multivariate analysis showed female urologists to administer IPSS and IIEF significantly less than their male counterparts (OR: 0.65; 95%CI: 0.38–1.08; OR: 0.61; 95%CI: 0.61; 0.42–0.89). Older and hospital physicians preferred IIEF (each: *p* < 0.001, Chi^2^-Test) both in bi- and multivariate analysis. The Karnofsky-Index was used significantly less often by females (*p* = 0.044, Chi^2^-Test). However, in multivariate analysis the gender difference was less prominent (OR: 0.76; 95%CI: 0.54–1.07). Males, older physicians, consultants and those in private practice applied AMS more frequently (males: *p* = 0.017; others: *p* < 0.001; Chi^2^-Test). In multivariate analysis, AMS administration was significantly higher in private practice (OR: 6.30; 95%CI: 4.58–8.66). Table 4 shows the results of the multivariate analysis.

**Table 4:** Factors influencing the choice of questionnaires used for HRQOL assessment (logistic regression analysis)

	IPSS OR (95% CI)	IIEF OR (95% CI)	Karnofsky-Index OR (95% CI)	AMS OR (95% CI)
Age^a^	0.95 (0.93–0.97)	0.96 (0.95–0.98)	1.01 (0.99–1.03)	0.99 (0.97–1.01)
Gender				
Male	1	1	1	1
Female	0.65 (0.38–1.08)	0.61 (0.42–0.89)	0.76 (0.54–1.07)	0.72 (0.48–1.08)
Working Environment				
Hospital	1	1	1	1
Private practice	1.10 (0.72–1.69)	0.67 (0.49–0.92)	0.78 (0.59–1.03)	6.30 (4.58–8.66)
Qualification				
Consultant	1	1	1	1
In Training	0.95 (0.39–2.33)	0.77 (0.41–1.43)	1.01 (0.59–1.72)	0.34 (0.14–0.81)

^a^In years as continuous variable

## Discussion

The principal aims of the study were to determine the importance of HRQOL in a clinical setting, to evaluate how useful, comprehensive and feasible clinicians find HRQOL assessment, to ascertain the most significant patient cohorts and aims for HRQOL assessment, and to describe the methods and instruments used.

Among our 1557 study participants, the attitude towards HRQOL was mainly positive, and most urologists considered HRQOL necessary as part of their clinical routine. The barriers towards the implementation of routine HRQOL assessment were constraints on time and financial resources. The length and complexity of questionnaires also had an adverse impact on HRQOL assessment. Our respondents regularly measured HRQOL in prostate cancer patients and patients with incontinence. Furthermore, they predominantly assessed HRQOL to determine a therapy, evaluate a follow-up or measure a baseline.

The central hypothesis of the survey was that HRQOL achieved an essential role in clinical practice if more than 30% of participants were employing validated questionnaires for recorded HRQOL assessment. These results suggest that the hypothesis was proven to be correct. While almost every respondent assessed HRQOL, more than 60 % employed validated questionnaires for recorded HRQOL assessment.

### General attitudes towards HRQOL and its clinical use

In general, urologists expressed interest in HRQOL assessment and were positive towards its clinical implementation, as had been found in other studies [5, 15, 16]. The positive image of HRQOL in urological guidelines [6] may have generated additional interest in the subject.

Most urologists agreed that the understanding of HRQOL among people might alternate. The WHO defines HRQOL as: ‘individuals’ perception of their position in life in the context of the culture and value systems in which they live and in relation to their goals, expectations, standards and concerns’ [1]. HRQOL can change at different stages of both disease and therapy as various concerns may arise [17].

HRQOL assessment enhances doctor-patient communication, facilitates the discussion of the psychosocial impact of the disease and ultimately improves the patient’s HRQOL [17–19]. In our study urologists perceived HRQOL assessment as valuable and suitable for daily use, while earlier surveys indicated that only a minority of physicians recognized the assessment of HRQOL as useful in clinical practice. Overall, there may have been a change of perception regarding HRQOL [2, 20].

In our study urologists felt confident to assess HRQOL, while in a different study pediatricians did not consider themselves to be sufficiently trained to assess HRQOL [15]. The different opinions may have been caused by the higher complexity of HRQOL assessment for children (including the intricacy of proxy methods) [21, 22].

The majority of urologists disapproved of the length and complexity of HRQOL questionnaires, while in another study physicians requested simplified scales for better applicability [16]. Notwithstanding, simplified scales may entail the risk of reductionism and the multidimensional construct of HRQOL could lose its significance. For an improved usability and easier HRQOL assessment in clinical routine, the employment of ten visual analogous scales was suggested [23].

Constraints of both time and financial resources were mentioned in our study. Nonetheless, these results did not differ much from previous findings [2, 24, 25]. However, the deficit of economic resources may be expedited by the impossibility of invoice for HRQOL assessment due to the German medical-fee schedule [26]. Consequently, to facilitate patient-centered care, the medical-fee schedule may have to be changed.

### Practical use of HRQOL in clinical routine

Urologists mostly assessed HRQOL in patients with prostate cancer, incontinence, and benign prostate hyperplasia. It may be that, out of all German urological guidelines, only those concerning these three conditions recommend strategies for HRQOL assessment [6]. Moreover, German urologists are likely to follow national guidelines, as has been proven in a recent survey [27]. Consequently, the enhancement of strategies for HRQOL assessment in guidelines for other diseases may aid HRQOL implementation.

HRQOL was assessed to support a therapy choice, create a baseline measure, and evaluate a follow-up status. It was least frequently evaluated for research purposes. This finding is especially interesting as most other studies have shown that HRQOL was primarily obtained for research purposes [5]. Supporting a therapy choice, creating a baseline measure, and evaluating a follow-up status are altogether important in patient-centered care. However, we believe that patients may benefit more from a continuous HRQOL assessment as proposed by Velikova et al. [17].

### Evaluation of recorded and verbal HRQOL assessment

The standardized, recorded HRQOL assessment is shown to influence doctor-patient communication positively and ultimately enhance the patient’s HRQOL [17–19]. It is evident that physicians must verbalize HRQOL and hence the differentiation of recorded and verbal approaches may seem academic. Consequently, less than 10 % used a recorded-only approach. However, it was important to investigate the use of standardized measures, which in turn are predominantly designed for recorded use. Most urologists used combined (verbal and written) HRQOL assessment.

Standardization of such a personal, individual and subjective measure as HRQOL raised skepticism among Wilm et al. [28], who argued that standardized measures would fail to incorporate individualized concepts of disease and bring a ‘scientific bias’ in approaching patients. Furthermore, proxy measures would raise unsolved methodological and ethical questions. Hence Wilm et al. advocated an open question: ‘How are you?’, to address HRQOL [28]. Our survey showed, however, that less than a quarter of urologists have exclusively asked this question.

Among numerous factors, doctor-patient communication is relationship based. Therefore, ‘How are you?’ is a question that may fail to address the multiple dimensions of HRQOL [29], whereas standardized HRQOL measurement proved to facilitate the doctor-patient relationship and, furthermore, enhance patients’ HRQOL [17–19].

An open question has reportedly failed to address important HRQOL issues, ascribed to a discrepancy in the topics of most importance to patients, who preferred to address social, psychological and spiritual issues, and doctors, who preferred to discuss the physical functioning and wellbeing [30]. Consequently, a standardized measure provides a chance to integrate all dimensions of HRQOL.

Wilson et al. investigated possible inadequacies of the standardized HRQOL measures [31]. However, contrary to Wilm et al., the Wilson group did not advise against their use but encouraged it in combination with an open discussion of HRQOL [31]. The same recommendations were given based on the results of other studies [16, 32].

### Used questionnaires

In our study, the IPSS had been the most frequently reported instrument used for HRQOL assessment. The German Society of Urology (DGU) recommends the use of IPSS for HRQOL assessment in patients with benign prostate hyperplasia [33]. It consists of a few symptom questions and a ‘bother score’ [34]. Although it is recommended for HRQOL assessment, it does not cover the psychosocial and spiritual dimensions of HRQOL, and hence important aspects of HRQOL may get lost. However, similarly to the ‘distress thermometer’ [35], clinicians could use the IPSS (and similar ‘bother scores’) as a screening for HRQOL impairment to decide whether to refer patients to a psychologist or a psycho-oncologist.

Compared to IPSS the EORTC-QLQ-C30 is a rather extensive HRQOL score. It is recommended by DGU for HRQOL assessment in patients with prostate cancer [36]. However, less than 5 % of our study participants have applied it in their clinical routine. More frequently, they reported using the Karnofsky Index and IIEF to assess HRQOL. However, these instruments are not capable of determining HRQOL. Similar to ECOG, the Karnofsky Index has been developed to evaluate general performance status [7, 37]. While these scores consist of single scales, the structure of IIEF is more complex. IIEF assesses Patient Reported Outcomes (PROs) related to erectile dysfunction using 15 Likert-scales. However, it does not measure the multidimensional concept of HRQOL [38]. These findings suggest that study participants may not have distinguished between Patient Reported Outcome Measures (PROMs) and HRQOL. Another study found that urologists were, in general, more accurate in recording sexual and incontinence symptoms (PROMs) than HRQOL [37].

PROMs and HRQOL are also frequently confused in the literature. For example, Doehn and Jocham discussed ECOG and the Karnofsky Index extensively in their review article on HRQOL assessment in urology, yet left unmentioned that both scores are incapable of measuring HRQOL [7]. Using the Karnofsky Index, urologists failed to detect significant role limitations [37].

Another example of the misrepresentation of HRQOL assessment is the recently published ‘Expanded Prostate Cancer Index Composite for Clinical Practice’ (EPIC-CP). It consists of 16 scales, each assessing the intensity of prostate-cancer-related symptoms [39]. Not a single scale evaluates psychosocial (or spiritual) aspects of the disease, hence failing to address the multidimensional concept of HRQOL as defined by the WHO [39].

### Clinical implications

Our findings are important for clinicians as they illustrate a typical pattern of clinical HRQOL assessment. The fact that over 60 % claim to assess HRQOL, while most of them use symptom-screening scales such as IPSS and some only ask an open question (‘How are you?’), is of particular importance for the clinical routine. Following either of the above strategies may lead to failure to assess the full spectrum of HRQOL [30, 34]. To avoid this, distinguishing separate PROMs from HRQOL is crucial. Furthermore, physicians tend to underestimate the impact of disease on patients’ HRQOL and hence should administer appropriate questionnaires [37].

We propose the use of validated instruments to investigate the impact of HRQOL on the disease, successional to an open discussion of HRQOL [16, 31, 32]. Consequently, along with Velikova et al., we recommend putting the emphasis of HRQOL assessment on the complaints that affect particular HRQOL dimensions according to the stages of chronic disease [17].

#### Importance and limitations

Primarily, HRQOL has achieved an essential role in clinical practice. This conclusion is supported by the fact that over 60 % of urologists reported frequent use of validated HRQOL questionnaires.

Nevertheless, a response bias may be a limitation, as respondents may have been more interested in the study topic than non-respondents [11]. Response bias can be calculated by estimating the difference between the demographics of respondents and non-respondents [12–14], as demographics can be associated with the attitude towards the survey topic. In our subgroup analysis, females were associated with lesser use of validated HRQOL instruments. However, this has not affected the response bias as no significant differences between the demographics of respondents and non-respondents were found.

A qualitative interrogation of non-respondents could have provided a better understanding of the non-respondents and helped to weigh the non-response bias. However, due to lack of fiscal and personnel resources such qualitative analysis could not be determined.

The use of a non-validated instrument could be considered a limitation. Therefore, ahead of the survey, the instrument’s feasibility was examined with two subsequent pre-tests. It was the first explorative study of its kind and the use of the questionnaire seems to be justified.

The survey was concluded nationwide, had a comparatively high response rate compared to other surveys among physicians and the socio-demographics of respondents did not differ significantly from non-respondents. Consequently, the chance of a response bias seems to be low.

Our survey addressed a general population of urologists and, therefore, the results could be considered generalizable, while other studies [3, 5] were based on rather specific populations and may have suffered from a selection bias.

To our knowledge, this is the first survey of German urologists on HRQOL in clinical routine. It provides detailed insights on the integration of HRQOL.

## Conclusions

Most urologists assess HRQOL in their daily clinical routine. Interestingly, the most ordinarily reported instruments were capable of rating symptoms, hence evaluating PROMs instead of measuring the complex concept of HRQOL. Conclusively, urologists’ knowledge concerning HRQOL assessment, analysis, and interpretation appears to be limited. To further integrate HRQOL into their clinical routine, urologists could benefit from a targeted education program.

## Abbreviations

AMS: Aging Male Symptoms
CI: Confidence interval
DGU: German Society of Urology
ECOG: East cooperative oncology group (Score)
EORTC-QLQ-C30: European Organization for Research and Treatment of Cancer Quality of Life Questionnaire for Cancer patients
EPIC-CP: Expanded Prostate Cancer Index Composite for Clinical Practice
HRQOL: Health related quality of life
ICIQ: International Consultation on Incontinence Questionnaire
ICSQoL: International Continence Society on Quality of Life
IIEF: International Index of Erectile Function
IPSS: International prostate syndrome score
OR: Odds ratio
PRO: Patient related outcome
PROM: Patient related outcome measure
WHO: World Health Organization

## References

[1] WHOQOL-Group (1997). WHOQOL: Measuring the Quality of Life.1;1–2.

[2] Morris J, Perez D, McNoe B (1998). The use of quality of life data in clinical practice. Qual Life Res.

[3] Young T, Maher J (1999). Collecting quality of life data in EORTC clinical trials—what happens in practice?. Psycho-Oncology.

[4] Davis K, Cella D (2002). Assessing quality of life in oncology clinical practice: a review of barriers and critical success factors. JCOM.

[5] Bossola M, Murri R, Onder G, Turriziani A, Fantoni M, Padua L (2010). Physicians’ knowledge of health-related quality of life and perception of its importance in daily clinical practice. Health Qual Life Outcomes.

[6] Schmick A. (2016). Attitudes of Urologists towards HRQOL and its Clinical Use. Doctoratethesis. University of Luebeck. 11–17.

[7] Doehn C, Jocham D (2002). Neues zur Lebensqualität in der urologischen Onkologie. Onkologie.

[8] Aaronson NK, Ahmedzai S, Bergman B (1993). The European Organization for Research and Treatment of cancer QLQ-C30: a quality-of-life instrument for use in international clinical trials in oncology. J Natl Cancer Inst.

[9] Osoba D, Rodrigues G, Myles J, Zee B, Pater J (1998). Interpreting the significance of changes in health-related quality-of-life scores. J Clin Oncol.

[10] Waldmann A, Schubert D, Katalinic A. Normative data of the EORTC QLQ-C30 for the German population: a population-based survey. PLoS One. 2013;8(9):e74149. https://doi.org/10.1371/journal.pone.0074149. 10.1371/journal.pone.0074149 PMC3769241 24058523

[11] Dillman DA (1991). The design and administration of mail surveys. Annu Rev Sociol.

[12] Kellerman S, Herold J (2001). Physician response to surveys a review of the literature. Am J Prev Med.

[13] McFarlane E, Olmsted M, Murphy J, Hill C (2007). Nonresponse bias in a mail survey of physicians. Eval Health Prof.

[14] van Goor H, Stuiver B (1998). Can weighting compensate for Nonresponse bias in a dependent variable? An Evaluation of weighting methods to correct for substantive bias in a mail survey among Dutch municipalities. Soc Sci Res.

[15] Baars RM, van der Pal SM, Koopman HM, Wit JM (2004). Clinicians’ perspective on quality of life assessment in paediatric clinical practice. Acta Paediatr.

[16] Skevington SM, Day R, Chisholm A, Trueman P (2005). How much do doctors use quality of life information in primary care? Testing the trans-theoretical model of behaviour change. Qual Life Res.

[17] Velikova G, Awad N, Coles-Gale R, Penny Wright E, Brown J, Selby P (2008). The clinical value of quality of life assessment in oncology practice - a qualitative study of patient and physician views. Psycho-Oncology.

[18] Velikova G, Booth L, Smith AB, Brown PM, Lynch P, Brown JM, et al. Measuring quality of life in routine oncology practice improves communication and patient well-being: a randomized controlled trial. J Clin Oncol. 2004;22(4):714–24. 10.1200/JCO.2004.06.078 14966096

[19] Detmar SB, Muller MJ, Schornagel NH, Wever LD, Aaronson NK (2002). Health-related quality of life assessments and patient-physician communication. JAMA.

[20] Bezjak A, Ng P, Skeel R, DePetrillo AD, Comis R, Taylor KM (2001). Oncologists' use of quality of life information: results of a survey of eastern cooperative oncology group physicians. Qual Life Res.

[21] Bianchini J, da Silva D, Nardo C, Carolino I, Hernandes F, Nardo N (2013). Parent-proxy perception of overweight adolescents' health-related quality of life is different according to adolescent gender and age and parent gender. Eur J Pediatr.

[22] Rotsika V, Coccossis M, Vlassopoulos M, Papaeleftheriou E, Sakellariou K, Anagnostopoulos D, et al. Does the subjective quality of life of children with specific learning disabilities (SpLD) agree with their parents' proxy reports? Qual Life Res. 2011;20(8):1271–8. 10.1007/s11136-011-9857-z 21308415

[23] Rosenzveig A, Kuspinar A, Daskalopoulou SS, Mayo NE (2014). Toward patient-centered care: a systematic review of how to ask questions that matter to patients. Baltimore Med.

[24] Straus SE, Sackett DL (1998). Getting research findings into practice: using research findings in clinical practice. Br Med J.

[25] Osoba D (2011). Health-related quality of life and cancer clinical trials. Ther Adv Med Oncol.

[26] Kassenärztliche Bundesvereinigung. (2016) EBM - Einheitlicher Bewertungsmaßstab; http://www.kbv.de/html/online-ebm.php; accessed: 5 Aug 2016.

[27] Fröhner M, Khan C, Koch R, Schorr S, Wirth M (2014). Implementierung der S3-Leitlinie Prostatakarzinom im klinischen Alltag. Urologe.

[28] Wilm S, Leve V, Santos S (2014). Is it quality of life that patients really want? Assessment from a general practitioner's perspective. Z Evid Fortbild Qual Gesundhwes.

[29] Arora NK, Jensen RE, Sulayman N, Hamilton AS, Potosky AL (2013). Patient-physician communication about health related quality of life problems: are non-Hodgkin lymphoma survivors willing to talk?. J Clin Oncol.

[30] Rodriguez KL, Bayliss N, Alexander SC (2010). How oncologists and their patients with advanced cancer communicate about health-related quality of life. Psychooncology.

[31] Wilson T, Birks Y, Alexander D (2013). Pitfalls in the interpretation of standardised quality of life instruments for individual patients? A qualitative study in colorectal cancer. Qual Life Res.

[32] Detmar SB (2003). Use of HRQOL questionnaires to facilitate patient–physician communication. Expert Rev Pharmacoecon Outcomes Res.

[33] Oelke M, Berges R, Tunn U (2009). Diagnostik und Differenzialdiagnostik des benignen Prostatasyndroms (BPS). AWMF.

[34] O’Leary M. (2005). Validity of the “bother score” in the Evaluation and treatment of symptomatic benign prostatic hyperplasia. Rev Urol.

[35] Zwahlen D, Hagenbuch N, Jenewein J, Carley M, Buchi S (2011). Adopting a family approach to theory and practice: measuring distress in cancer patient–partner dyads with the distress thermometer. Psycho-Oncology.

[36] Guideline Programme Oncology (German Society of Oncology, German Cancer Aid, AWMF): Interdisciplinary guideline of S3 quality for early detection, diagnosis and therapy of different stages of prostate cancer. Short version (German), 4.0, 2016, *AWMF* Registernumber:043/022OL,http://leitlinienprogramm-onkologie.de/Prostatakarzinom.58.0.html (accessed on 11 Mar 2017).

[37] Litwin MS, Lubeck DP, Henning JM, Carroll PR (1998). Differences in urologist and patient assessments of health related quality of life in men with prostate cancer: results of the CaPSURE database. J Urol.

[38] Rosen RC, Riley A, Wagner G, Osterloh IH, Kirkpatrick J, Mishra A (1997). The international index of erectile function (IIEF): a multidimensional scale for assessment of erectile dysfunction. J Urol.

[39] Chang P, Szymanski KM, Dunn RL (2011). Expanded prostate cancer index composite for clinical practice: development and validation of a practical health related quality of life instrument for use in the routine clinical care of patients with prostate cancer. J Urol.

